# Neuroprotective Effect of Echinacoside in Subacute Mouse Model of Parkinson's Disease

**DOI:** 10.1155/2019/4379639

**Published:** 2019-01-30

**Authors:** Yan Liang, Chang Chen, Baomei Xia, Wei Wu, Juanjuan Tang, Qing Chen, Lili Tang, Hui Yang, Zhennian Zhang, Yan Lu, Ye Yang, Yang Zhao

**Affiliations:** ^1^Nanjing University of Chinese Medicine, Nanjing, Jiangsu, China; ^2^Department of Neurology, The Third Affiliated Hospital of Nanjing University of Chinese Medicine, Nanjing, Jiangsu, China; ^3^Faculty of Rehabilitation Science, Nanjing Normal University of Special Education, Nanjing, Jiangsu, China; ^4^Physiology Research Section, School of Medicine and Life Sciences, Nanjing University of Chinese Medicine, Nanjing, Jiangsu, China; ^5^Department of Neurology, Nanjing Pukou Hospital of Traditional Chinese Medicine, Nanjing, Jiangsu, China; ^6^Center for Modernization of Chinese Medicine and Database, The Third Affiliated Hospital of Nanjing University of Chinese Medicine, Nanjing, Jiangsu, China

## Abstract

**Objective:**

To study the protective effect of Echinacoside for 1-methyl-4-phenyl-1,2,3,6-tetrahydropyridine (MPTP) induced dopaminergic (DA) neurons injury in the subacute mouse model of Parkinson's disease (PD) and to explore its mechanism of action.

**Methods:**

We chose 10 weeks of healthy wild type C57BL/6 male mice, hypodermic MPTP 30 mg/kg/day, five days, to prepare PD subacute mouse model. Behavior indexes of open field test and pole test were applied to examine the function of ECH to PD subacute mice model of PD sample action. The effects of ECH on dopaminergic neurons and astrocyte were examined using Immunohistochemistry including tyrosine hydroxylase (TH) and glial fibrillary acidic protein (GFAP) expression. The total numbers of TH-positive neurons and GFAP-positive cells in the substantia nigra pars compacts (SNpc) and ventral tegmental area (VTA) were obtained stereologically using the optical fractionator method. Enzyme-linked immunosorbent assay (ELISA) method was used to detect the inflammatory cytokines in the serum, including TNF-*α* (Ttumor necrosis factor alpha) and IFN-*γ* (interferon gamma). Protein expressions of ionized calcium binding adaptor molecule 1 (IBA-1), TNF-*α*, Cleaved caspase-3, glial derived neurotrophic factor (GDNF), and phosphorylated and total extracellular signal-regulated kinase (p-ERK and ERK) in the anatomical region of substantia nigra (SN) were tested by protein immunoblot method (i.e., Western blotting).

**Results:**

ECH reversed the reduction of total distance in open field test in MPTP-induced PD model mice (P < 0.01), shortened the return time and total time of PD subacute model mice in pole test (P < 0.01, P < 0.05), significantly reversed the reduction of TH positive neurons induced by MPTP (P < 0.05), and reduced the activation of astrocytes (P < 0.05). Meanwhile, ECH significantly inhibited the expression of IBA-1, Cleaved caspase-3, and TNF-*α* in midbrain of MPTP model mice (P < 0.05, P < 0.05, and P < 0.05) and upregulated the expression of GDNF (P < 0.05). And ECH lowered the level of TNF-*α* and IFN-*γ* in serum (P < 0.05, P < 0.05).

**Conclusion:**

ECH has protective effects on the MPTP subacute model mice, its mechanism may be through inhibiting activation of microglia and astrocytes, reducing inflammatory reaction and promoting the secretion of neurotrophic factors, and eventually resulting in the reduction of the DA neurons apoptosis.

## 1. Introduction

Parkinson's disease (PD) is the second most common age-related neurodegenerative disease, which is characterized by progressive loss of dopaminergic (DA) neurons and the appearance of Lewy body in the cytoplasm in the midbrain substantia nigra compacta. Chronic neuroinflammation is also one of the signs of PD pathophysiology. The postmortem analysis of human PD patients and experimental animals showed that activation of glial cells and increased levels of proinflammatory cytokines are common features of PD brain [1, 2]. Previous study showed that activation of astrocytes and microglia due to the release of promoting inflammatory cell factor aggravated the degeneration of DA neurons in the SNpc [3]. Therefore, the inflammatory process is considered to be a promising intervention target for PD and other neurodegenerative diseases. Echinacoside (ECH) is derived from the fleshy stems of* Cistanche deserticola*. Its pharmacological effects involve skeleton protection, liver protection, antioxidation, anti-inflammation, antiaging [4]. With the deepening and development of pharmacology, a growing number of studies have been focused on the role of ECH in the treatment of neurological diseases in recent years [5–7]. The purpose of this study was to establish a subacute PD model of MPTP in vivo by using C57BL/6 mice, in which Selegiline was used as the positive drug control to study the neuroprotective effect of ECH on PD model mice and its underlying mechanism of action.

## 2. Materials and Methods

### 2.1. Animals and Groups

Experiments were conducted using male C57BL/6J mice at 10 weeks of age and weighing 25 to 30g. The animals were maintained in standard conditions (12/12h light/dark cycle, 22±2°C, and relative humidity of 55±5%) and allowed access to food and water ad libitum. All animal procedures conformed to the Guide for the Care and Use of Laboratory Animals and were approved by the Institutional Animal Care and Use Committee at Nanjing University of Chinese medicine. The experimenters were blinded to the assignments of the mice. Before the experiment, the animals were housed in our facilities for two weeks to acclimate; then the animals were randomly divided into five groups (10 mice in each group): Saline control group, model group (MPTP), MPTP+ECH group, positive drug control group (MPTP+Selegiline), and ECH group.

### 2.2. Drugs and Reagents

Echinacoside (HPLC≥98%,82854-37-3) was purchased from Chengdu Research Institute of Biology of the Chinese Academy of Sciences, China. Selegiline was purchased from Orion, Finland. 1-Methyl-4-phenyl-1,2,3,6-tetrahydropyridine hydrochloride (MPTP, M0896) and anti-tyrosine hydroxylase (TH) antibody (T1299) were purchased from Sigma, America. Antiglial fibrillary acidic protein (GFAP) antibody (MAB360) were purchased from Millipore, America. Diaminobenzidine (DAB, AR1000) was purchased from Boster Biological Technology Co. Ltd., America. ELISA Kits (SBJ-M0030, SBJ-M0038) were purchased from Nanjing SenBeiJia Biological Technology Co. Ltd. The primary antibodies used in the study were as follows: Anti-ionized calcium binding adaptor molecule 1 (IBA-1) antibody (016-20001, Wako, Japan), anti-TNF-*α* antibody (bs-0078R, Beijing Biosynthesis Biotechnology Co. Ltd., China), anti-Cleaved Caspase-3 antibody (9664s, Cell Signaling Technology, America), antiglial derived neurotrophic factor (GDNF) antibody (sc-328, Santa cruz, America), antiphosphorylated and total extracellular signal-regulated kinase (p-ERK and ERK) antibody (4370, 4695, Cell Signaling Technology, America), and anti-*β*-Tublin antibody (10094-1-AP, Proteintech Group, America). The secondary antibodies used in the study were as follows: HRP-labeled Goat anti-Rabbit IgG and HRP-labeled Goat anti-mouse IgG (ZB-2301, ZB-2305, Beijing Zhongshan Jinqiao Biotechnology Co., Ltd., China).

### 2.3. Administration of Drugs

The animals were given ECH 30mg/kg, Selegiline 1mg/kg, or the same volume of saline gavage at 10:00am per day for 7 days before the beginning of model-making process. MPTP (30mg/kg) was injected subcutaneously to the model-making animals at 9:00am from day 8d to day 12d, and ECH or Selegiline was given gavage administration after 1h, while non-model-making animals were given subcutaneously or gavage treatment with saline. From 13d to 16d, ECH or Selegiline was still given to the model-making animal at 10:00am daily, while non-mold-making animals were given saline gavage. At 15:00pm on day 16, behavioral tests were conducted on all the groups of animals. At 10:00am on day 17, all the animals were sacrificed after orbital blood extraction, 4 mice of each group were perfused via intracardial infusion with saline (0.9%) followed by 4% paraformaldehyde (PFA), and the brains were collected. In each group, another 6 animals' tissues of ventral midbrain and striatum were dissected rapidly on ice frozen in liquid nitrogen and stored at -80°C until used.

### 2.4. Locomotor Activity

The open-field test can be used to observe and evaluate the spontaneous activity of mice. The test device consists of two parts: the open-field universal sound insulation box and the automatic data collection and processing system. The open-field reaction box is an organic glass box (40cm long, 40cm wide, and 15cm high), and the test area is well illuminated. The experiment was carried out in a quiet environment. Put the animal into the center of the bottom of the box and turn on the camera to observe for 5 min. The automatic data acquisition device sends out infrared ray to track all the trajectories of the random movement in the mouse box, which is total distance. Before the test, 75% ethanol was used to thoroughly wipe the inner wall and bottom surface of the box, so as not to affect the results of the next test. Replace the animals and continue the experiment.

### 2.5. Pole Test

Pole test can be used to assess the coordination and balance ability of mice, and it was conducted at 15:00pm on day 16. According to Chen's protocol [8], the PV tube with a length of 55cm and a diameter of 1cm was selected. The outside of the PV tube was tightly wrapped with white tape to prevent slip. The PV tube was fixed on the plastic foam base and placed in the cage and the base was covered with dressing. The upper end of the tube was covered with a spherical protruding point with a diameter of 2cm, which was used as the attachment point of the mouse during the experiment. The mouse was placed on the spherical protruding point with its head upward during the test, and the time from the top of the placed bar to the head to turn down (t-turn) and the total time from the top of the placed bar to climb to the bottom of the tube to land (t-total) were recorded. Each mouse was tested 3 times, with an interval of 2min between each test, and an average value of the 3 times was taken as the data.

### 2.6. Immunohistochemical Staining [8]

After perfusion from the left ventricle, the brains of mice were rapidly removed and placed in 4% paraformaldehyde to be fixed overnight. Sucrose solution was used for gradient dehydration and OCT gel was used for embedding. We prepared brain tissue sections (30 microns) with frozen slicer and chose SN-VTA slices in line with the atlas of Paxinos and Franklin [9]. Sections were blocked for 1h and incubated with anti-TH primary antibody (1: 1000) and anti-GFAP primary antibody (1: 1000) for 12h. After washing there times in 0.01M PBS, the slices were incubated with the secondary antibody of goat anti-mouse IgG H&L (HRP) (1:2000) for 2h. After washing the brain slices, a modification of the ABC system (Kit Vectastain ABC, Vector Laboratory Inc.) was used to detect the antibody complex. Image-Pro Express 6 (Media Cybernetics) was applied to stereologically quantify the number of TH positive neurons and GFAP positive astrocytes, and the mean number of them in each brain was picked up through the definite quantity of four alternating sections. Olympus camera (DP72; Olympus) was used to take images at an original magnification of 100×.

### 2.7. Western Blotting

Tissues of substantia nigra were lysed in RIPA buffer containing protease inhibitors and phosphatase inhibitors. Protein concentration was determined colorimetrically by NANODROP 2000 (Thermo Scientific). Protein lysates were separated by 10% SDS-PAGE electrophoresis and were transferred onto polyvinylidenedifluoride (PVDF) membranes. After blocking with 5% BSA for 1h, the following antibodies were used: Anti-IBA-1 antibody (1:300), anti-TNF-*α* antibody (1:200), anti-Cleaved Caspase-3 antibody (1:200), anti-GDNF antibody (1:200), anti-phosphorylated-ERK (1:500) antibody and anti-ERK antibody (1:500), anti-*β*-Tublin antibody (1:2000). After the blots were incubated with antibodies overnight at 4°C, they were incubated with horseradish peroxidase-conjugated secondary antibodies for 1h. The blots were visualized using the Super Signal West Pico Chemiluminescent Substrate (Thermo Fisher Scientific Inc.). The resulting data was analyzed statistically using SPSS Statistics 20. All experiments were performed in triplicate. The final data are expressed as a ratio of the relative optical density (ROD) of the protein of interest to the ROD of *β*-tubulin. A p value < 0.05 was considered significant.

### 2.8. Enzyme-Linked Immunosorbent Assay

The blood of each mouse was taken out 1.5ml to be centrifuged, under the condition of 4°C, 3000 r/min, and 10 cm centrifugal radius, for 10 min, and then the blood was stored in 20°C. The contents of inflammatory factors such as TNF-*α* and IFN-*γ* were detected by enzyme-linked immunoassay kit. All the operations are carried out in accordance with the instruction of kits strictly.

### 2.9. Statistical Analysis

SPSS Version 20.0 (IBM) was used for all statistical analyses. All experiments were performed at least three times. Comparisons between two groups were performed using a two-sample Student's t-test, and comparisons between multiple groups were performed using a one-way ANOVA followed by post hoc analysis of Tukey's HSD and LSD multiple comparisons test. All data are presented as mean ± SEM, and statistical significance was accepted at the 5% level unless otherwise indicated.

## 3. Result

### 3.1. Effect of ECH on Behavior Test in Subacute PD Model Mice

#### 3.1.1. Locomotor Activity

**Figure 1:**
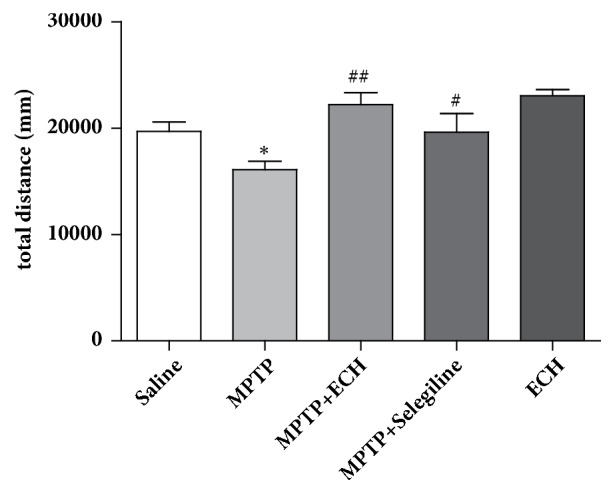
Total distance of open field test. Saline means saline control group, MPTP means model group, MPTP+ECH means MPTP+ECH group, MPTP+Selegiline means positive drug control group, and ECH means ECH group. *∗*p<0.05, compared with Saline; #p<0.05 and ##p<0.01, compared with MPTP.

In the open field test, the total distances of activity in MPTP model group mice was significantly shortened, and the difference was statistically significant compared with that in saline control group (P<0.05). While compared with the model group, the total distance of activity in MPTP+ECH group was significantly longer (P<0.01) (Figure 1).

#### 3.1.2. Equilibrium Coordination

**Figure 2:**
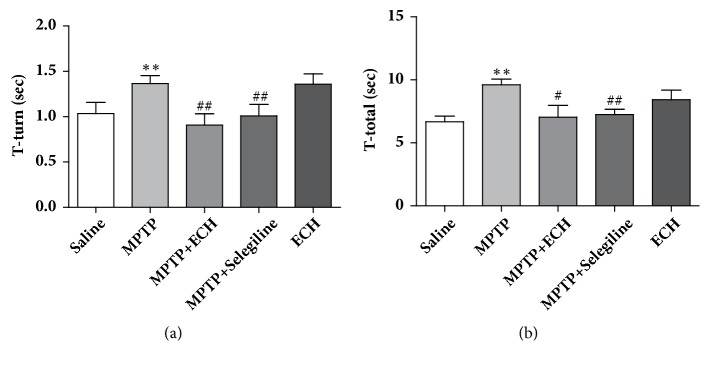
Pole test. (a) Time of return. (b) Time of total. Saline means saline control group, MPTP means model group, MPTP+ECH means MPTP+ECH group, MPTP+Selegiline means positive drug control group, and ECH means ECH group. *∗∗*p<0.01, compared with saline; #p<0.05 and ##p<0.01, compared with MPTP.

In the pole test, the return time of mice in the MPTP model group was significantly longer than that of saline control group (P<0.01); while compared with the model group, the time of MPTP+ECH group was shorter, and the difference was statistically significant (P < 0.01). As to the total time, it was significantly increased in the model group compared to saline control group (P < 0.01); meanwhile it was significantly shortened in the MPTP+ECH group compared to the model group (P < 0.05) (Figure 2).

### 3.2. Effects of ECH on Dopaminergic Neurons in the Midbrain of Subacute PD Model Mice

**Figure 3:**
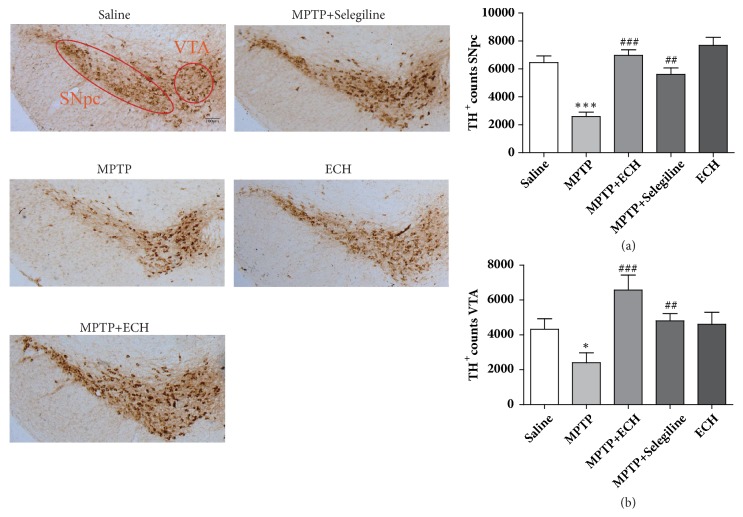
Representative microphotographs of dopaminergic neurons stained for TH and the quantification of TH positive cell in each group. The pictures were taken at an original magnification of 10×. Scale bars: 100*μ*m. Saline means saline control group, MPTP means model group, MPTP+ECH means MPTP+ECH group, MPTP+Selegiline means positive drug control group, and ECH means ECH group. *∗*p<0.05, *∗∗∗*p<0.001, compared with saline; ##p<0.01 and ###p<0.001, compared with MPTP.

Tyrosine hydroxylase is the specific markers for dopaminergic neurons; the number of TH positive cells can be used as the number of DA neurons. The stereo counting showed that the TH positive cells number of the midbrain in the MPTP model group decreased by 63.1% compared with the saline control group, in which the number in SNpc area decreased by 59.9%, and the number in the VTA area decreased by 44.6%. Both differences were significant compared with the control group (P<0.001, P<0.05). While in the ECH treatment group, the number of TH positive cells in both SNpc and VTA increased observably, which was statistically significant compared with the model group (P < 0.001, P < 0.001) (Figure 3).

### 3.3. Effects of ECH on Astrocytes in the Midbrain of Subacute PD Model Mice

**Figure 4:**
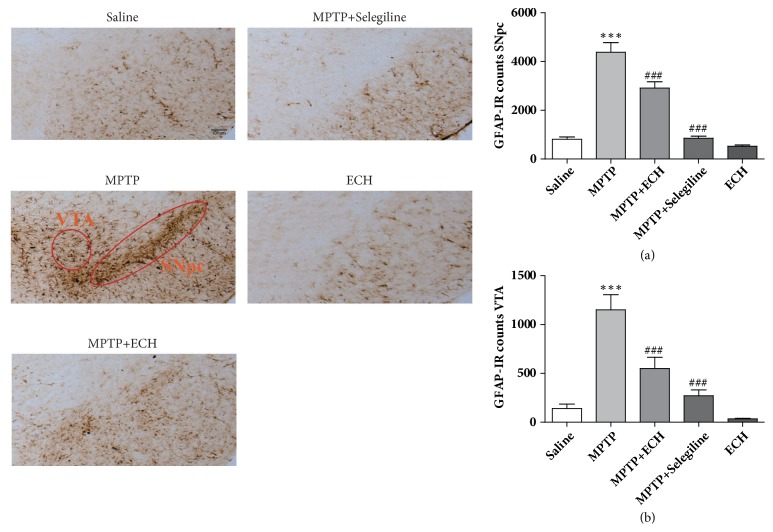
Representative microphotographs of dopaminergic neurons stained for GFAP and the quantification of GFAP positive cell in each group. The pictures were taken at an original magnification of 10×. Scale bars: 100*μ*m. Saline means saline control group, MPTP means model group, MPTP+ECH means MPTP+ECH group, MPTP+Selegiline means positive drug control group, and ECH means ECH group. *∗∗∗*p<0.001, compared with saline; ###p<0.001, compared with MPTP.

As a marker of activation in astrocyte, GFAP can be used to detect the number of active astrocytes. According to the results of immunohistochemistry, the astrocytes in model group mice were activated in a pattern of hypertrophy, and the number of active astrocytes was significantly increased in both SNpc area and VTA area compared with saline control group (P < 0.001, P < 0.001). After the ECH treatment, the morphology of astrocytes were restored to the saline control group, and the number of GFAP positive cells in SNpc and VTA area was significantly decreased (P < 0.001, P < 0.001). (Figure 4)

### 3.4. Effects of ECH on Serum Inflammatory Factors in Subacute PD Model Mice

**Figure 5:**
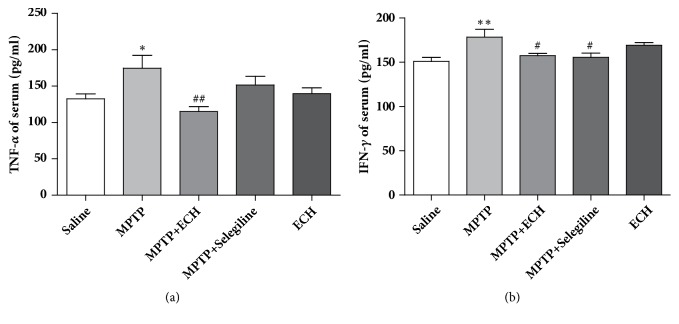
Alteration of TNF-*α* and IFN-*γ* in serum of mice. Saline means saline control group, MPTP means model group, MPTP+ECH means MPTP+ECH group, MPTP+Selegiline means positive drug control group, and ECH means ECH group. *∗*p<0.05 and *∗∗*p<0.01, compared with saline; #p<0.05 and ##p<0.01, compared with MPTP.

Enzyme-linked immunosorbent assay showed that the concentrations of inflammatory factors including TNF-*α* and IFN-*γ* in the serum of model group mice were significantly higher than that of saline control group (P < 0.05; P < 0.01). After the treatment of ECH, the concentrations of the two factors were significantly reduced (P < 0.01; P < 0.05) (Figure 5).

### 3.5. Effects of ECH on the Expression of Midbrain Proteins Including IBA-1, Cleaved Caspase-3, GDNF, TNF-*α*, p-ERK, and ERK in Subacute PD Mice

**Figure 6:**
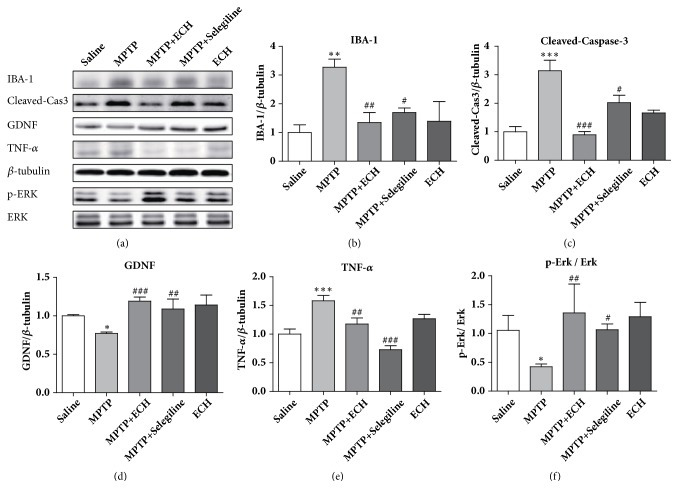
Protein expression of IBA-1, Cleaved-Caspase-3, GDNF, TNF-*α*, p-ERK, and ERK. Saline means saline control group, MPTP means model group, MPTP+ECH means MPTP+ECH group, MPTP+Selegiline means positive drug control group, and ECH means ECH group. *∗*p<0.05, *∗∗*p<0.01, *∗∗∗*p<0.001, compared with saline; #p<0.05, ##p<0.01, and ###p<0.001, compared with MPTP.

Western blotting analysis showed that MPTP induced a marked IBA-1, Cleaved Caspase-3, and TNF-*α* protein increase (P<0.01, P<0.001, and P<0.001), while the expression of GDNF and ERK phosphorylation level was significantly decreased (P<0.05, P<0.05) in midbrain tissue compared to saline control group. After the ECH administration, the expressions of IBA-1, Cleaved Caspase-3, and TNF-*α* were significantly decreased (P < 0.01, P < 0.001, and P < 0.01), and the expressions of GDNF and the phosphorylation level of ERK were significantly increased (P < 0.001; P < 0.01). (Figure 6)

## 4. Discussion

Parkinson's disease is a common central nervous system degeneration disease in the elderly. Its main clinical features are not only bradykinesia, static tremor, myotonia, and abnormal posture, but also olfactory disorder, constipation, insomnia, fatigue, depression, and anxiety and other nonmotor symptoms [10, 11]. The pathogenesis of PD is complex, which may be caused by aging, genetic, and environmental factors. The pathological change of SNpc DA neuron degeneration lost and appearance of Lewy body may be connected with cell autophagy, proteasome dysfunction, mitochondrial function defect, oxidative stress and immune response, inflammation, excitatory neurotoxicity, and other complex mechanisms [10, 12]. Currently, dopaminergic drug therapy is the major treatment of PD. Neither supplementing the DA neurotransmitter nor activating DA receptors can reverse the loss of DA neurons and the appearance of Lewy body. That means the current PD treatment plan is mainly for the symptoms without affecting the progress of the disease [13]. Therefore, there has been increasing interests in studying agents like ECH that affect the mechanistic processes of PD as novel treatment. This study specifically found that (1) ECH could improve the PD-like behavior of subacute model mice induced by MPTP and the loss of DA neurons in the substantia nigra of model mice was saved, which is similar to the reports of Qing Zhao [14]. (2) ECH could also reverse against the astrocyte overactivation, microglia activation, serum, and mesencephalic inflammatory factors in midbrain induced by MPTP in PD subacute model mice. (3) ECH was able to increase the expression of GDNF and ameliorate the overexpression of MPTP-induced IBA-1 and Cleaved Caspase-3 and the decrease of phosphorylated ERK.

MPTP is the most commonly used environmental toxin for the preparation of PD animal model, which is highly fat-soluble and extremely easy to pass through the blood brain barrier. MPTP is metabolized by monoamine oxidase in astrocytes and turned into ionized 1-methyl-4-phenyl pyridine (MPP^+^), transferred by the Dopamine transporter (DAT) to the DA neurons. MPTP produces obvious pathological and biochemical damage of DA neurons in C57BL/6J mice, so it has a great advantage over other PD models in terms of efficacy evaluation. We prepared subacute PD models in C57BL/6J mice by MPTP (subcutaneous injection) for five consecutive days and observe that MPTP can successfully induce animal PD behavior, cause the loss of DA neurons by immune histochemical and behavior testing.

Echinacoside is the key components in the identification and assaying of herba cistanche in Chinese pharmacopoeia. In recent years, it has been found that ECH can cross the blood brain barrier [15] and has a good protective effect on the central nervous system. According to Zhao's research [14], we chose the optimal therapeutic does of 30mg/kg ECH as an intervention, the behavior of MPTP-induced animal models improved markedly, the DA neurons deaths dropped significantly, the expression of Cleaved Caspase-3 decreased obviously, and all the above indicated ECH could protect DA neurons of PD model mice by antiapoptotic effect. This is similar to Zhao's report [14]. The difference between two experiments lies in Zhao detected apoptosis related protein directly, but Zhao has not studied how MPTP causes apoptosis of DA neurons and how ECH works against apoptosis. Therefore, this study explored the mechanism of MPTP-induced apoptosis of DA neurons and the role of ECH in neurorescue based on glial cells.

Microglia is an immune cell in the brain; when activated, it produces and releases proinflammatory cytokines that cause damage to DA neurons. Inflammatory factors such as IFN-*γ*, IL-1*β*, TNF-*α*, IL-2, and IL-6 [1] are founded in the SN, striatum, and cerebrospinal fluids of autopsy in patients with PD; meanwhile, the expressions of TNF-*α* receptors R1 (p55), bcl-2, soluble Fas, caspase-1, and caspase-3 are increased [16]. Therefore, proinflammatory cytokines activated and released by microglia-mediated factor may be the one reason of death of DA neurons. Astrocytes secrete a variety of neurotrophic factors, of which GDNF is particularly important, because it can specifically promote the repair of DA neurons in injured midbrain, and it is considered as the most promising nutrient factor in PD treatment. According to reports in the literature [17], significant improvement can be observed after astrocytes which are carrying the high expression of GDNF gene were implanted into the SN region of animal models of PD. The treatment of GDNF depends on the ERK signaling pathway, which promotes cell survival [18]. It has been reported that application of phosphatidylinositol-related D1-like receptor agonists in rat astrocytes can enhance the expression of phosphorylated ERK and promote the migration of astrocytes [19]. GDNF can activate ERK and induce the upregulation of its own expression in SH-SY5Y cells and rat VTA region [20, 21]. At the same time, ERK signaling pathway also plays a key role in the inflammatory pathological changes of PD, and their coeffects may affect the pathogenesis of PD and the progression of the disease. In the resting state, ERK/MAPK are located in the cytoplasm of the cells. When activated, ERK is rapidly phosphorylated. Then, activated ERK enters the nucleus to regulate the activities of other transcription factors, which make the change of gene transcription impact the ascension or decline of the expression of respective protein gene and finally cause the changes in cell's energy metabolism, physiological, and biochemical function [22]. This study found that ECH can upregulate the phosphorylation of ERK, increase the expression of GDNF, and significantly downregulate the activation of microglia-IBA-1. Therefore, we believe that ECH exerts neurotrophic effects by upregulating the phosphorylation level of ERK, increasing the expression of GDNF, and at the same time inhibiting the activation of microglia and astrocytes, and further reducing the content of inflammatory factor in serum and midbrain, which restrains DA neuronal apoptosis.

The antiapoptotic effect of ECH has been confirmed. To our knowledge, it is the first time to explore the signal pathways of anti-inflammatory and antiapoptosis and anti-inflammatory effects of ECH. The expression of GDNF might be related to the regulation of ERK signaling pathway. The results of this experiment enriched and extended the protective mechanisms of ECH in PD, providing useful targets for PD therapeutics development and also the related basic research. In the past ten years, there have been a large number of experimental studies on traditional Chinese medicine compound and monomer that have been confirmed with the protective effects on DA neurons in PD. On this basis, exploring and confirming the targets of these drugs and their neuroprotective mechanisms can provide a theoretical basis for the clinical treatment of PD and drug screening.

## Data Availability

The data used to support the findings of this study are available from the corresponding author upon request.

## References

[1] de Lella Ezcurra A. L., Chertoff M., Ferrari C., Graciarena M., Pitossi F. (2010). Chronic expression of low levels of tumor necrosis factor-alpha in the substantia nigra elicits progressive neurodegeneration, delayed motor symptoms and microglia/macrophage activation. *Neurobiology of Disease*.

[2] Booth H. D. E., Hirst W. D., Wade-Martins R. (2017). The Role of Astrocyte Dysfunction in Parkinson's Disease Pathogenesis. *Trends in Neurosciences*.

[3] Wang Q., Liu Y., Zhou J. (2015). Neuroinflammation in Parkinson's disease and its potential as therapeutic target. *Translational Neurodegeneration*.

[4] Lei L., Yang F. Q., Zhang T. Y., Tu P., Wu L., Ito Y. (2001). Preparative isolation and purification of acteoside and 2′-acetyl acteoside from *Cistanches salsa* (C.A. Mey.) G. Beck by high-speed counter-current chromatography. *Journal of Chromatography A*.

[5] Chen W., Lin H.-R., Wei C.-M. (2017). Echinacoside, a phenylethanoid glycoside from Cistanche deserticola, extends lifespan of Caenorhabditis elegans and protects from A*β*-induced toxicity. *Biogerontology*.

[6] Shiao Y.-J., Su M.-H., Lin H.-C., Wu C.-R. (2017). Echinacoside ameliorates the memory impairment and cholinergic deficit induced by amyloid beta peptides via the inhibition of amyloid deposition and toxicology. *Food & Function*.

[7] Zhang Y., Long H., Zhou F. (2017). Echinacoside's nigrostriatal dopaminergic protection against 6-OHDA-Induced endoplasmic reticulum stress through reducing the accumulation of Seipin. *Journal of Cellular and Molecular Medicine*.

[8] Chen C., Xia B., Tang L. (2018). Echinacoside protects against MPTP/MPP+-induced neurotoxicity via regulating autophagy pathway mediated by Sirt1. *Metabolic Brain Disease*.

[9] Paxinos G., Keith B. J. F. (2004). *The mouse brain in stereotaxic coordinates*.

[10] Kalia L. V., Lang A. E. (2015). Parkinson's disease. *The Lancet*.

[11] Postuma R. B., Berg D., Adler C. H. (2016). The new definition and diagnostic criteria of Parkinson's disease. *The Lancet Neurology*.

[12] Calabresi P., Di Filippo M. (2015). Multitarget disease-modifying therapy in Parkinson's disease?. *The Lancet Neurology*.

[13] Valera E., Masliah E. (2016). Therapeutic approaches in Parkinson's disease and related disorders. *Journal of Neurochemistry*.

[14] Zhao Q., Gao J., Li W., Cai D. (2010). Neurotrophic and neurorescue effects of Echinacoside in the subacute MPTP mouse model of Parkinson's disease. *Brain Research*.

[15] Zhu M., Lu C., Li W. (2013). Transient exposure to echinacoside is sufficient to activate Trk signaling and protect neuronal cells from rotenone. *Journal of Neurochemistry*.

[16] Gordon R., Anantharam V., Kanthasamy A. G. (2012). Proteolytic activation of proapoptotic kinase protein kinase Cdelta by tumor necrosis factor alpha death receptor signaling in dopaminergic neurons during neuroinflammation. *Journal of Neuroinflammation*.

[17] Drinkut A., Tereshchenko Y., Schulz J. B., Bähr M., Kügler S. (2012). Efficient gene therapy for Parkinson's disease using astrocytes as hosts for localized neurotrophic factor delivery. *Molecular Therapy*.

[18] Knapska E., Kaczmarek L. (2004). A gene for neuronal plasticity in the mammalian brain: Zif268/Egr-1/NGFI-A/ Krox-24/TIS8/ZENK?. *Progress in Neurobiology*.

[19] Huang C., Wu J., Liao R., Zhang W. (2012). SKF83959, an Agonist of Phosphatidylinositol-Linked D1-Like Receptors, Promotes ERK1/2 Activation and Cell Migration in Cultured Rat Astrocytes. *PLoS ONE*.

[20] He D.-Y., Ron D. (2006). Autoregulation of glial cell line-derived neurotrophic factor expression: implications for the long-lasting actions of the anti-addiction drug, Ibogaine. *The FASEB journal : official publication of the Federation of American Societies for Experimental Biology*.

[21] Barcia C. (2013). Glial-Mediated Inflammation Underlying Parkinsonism. *Scientifica*.

[22] Kim E. K., Choi E.-J. (2015). Compromised MAPK signaling in human diseases: an update. *Archives of Toxicology*.

